# Brain metastases from breast cancer using magnetic resonance imaging: A systematic review

**DOI:** 10.1002/jmrs.715

**Published:** 2023-08-10

**Authors:** Mahdi Mohammadi, Sana Mohammadi, Hojatollah Hadizadeh, Mahsa Olfati, Fatemeh Moradi, Ghazal Tanzifi, Sadegh Ghaderi

**Affiliations:** ^1^ Department of Medical Physics and Biomedical Engineering, School of Medicine Tehran University of Medical Sciences Tehran Iran; ^2^ Department of Medical Sciences, School of Medicine Iran University of Medical Sciences Tehran Iran; ^3^ Department of Radiology and Nuclear Medicine, School of Paramedical Kermanshah University of Medical Sciences Kermanshah Iran; ^4^ Department of Energy Engineering & Physics Amirkabir University of Technology (Tehran Polytechnic) Tehran Iran; ^5^ Department of Nuclear Engineering Islamic Azad University, Central Tehran Branch Tehran Iran; ^6^ Department of Neuroscience and Addiction Studies, School of Advanced Technologies in Medicine Tehran University of Medical Sciences Tehran Iran

**Keywords:** Brain metastases, brain metastasis, breast cancer, magnetic resonance imaging

## Abstract

Despite improvements in imaging and treatment approaches, brain metastases (BMs) continue to be the primary cause of mortality and morbidity in about 20% of adult cancer patients. This research aimed to review the magnetic resonance imaging (MRI) and clinical characteristics of BMs resulting from breast cancer (BC). A systematic review of original research articles published from January 2000 to June 2023. We selected studies that reported MRI findings of BMs in BC patients. We excluded reviews, case reports, books/book chapters, animal studies and irrelevant records. We identified 24 studies that included 1580 BC patients with BMs. T1‐weighted (T1‐w) (pre‐ and postcontrast), T2‐weighted (T2‐w), fluid‐attenuated inversion recovery (FLAIR) and T2*‐weighted (T2*‐w) was used to measure the lesion size, shape and area. In other studies, advanced structural techniques including diffusion‐weighted imaging (DWI), diffusion tensor imaging (DTI) and susceptibility‐weighted imaging (SWI) were used to more precisely and sensitively evaluate the pathological area. Furthermore, functional and metabolic techniques like functional MRI (fMRI), magnetic resonance spectroscopy (MRS) and perfusion‐weighted imaging (PWI) have also been utilised. The MRI findings of BMs varied depending on the MRI technique, the BC subtype, the lesion size and shape, the presence of haemorrhage or necrosis and the comparison with other brain tumours. Some MRI findings were associated with prognosis, recurrence or cognitive impairment in BC patients with BMs. MRI detects, characterises and monitors BMs from BC. Findings vary by MRI technique, BC subtype, lesion characteristics and comparison with other brain tumours. More research should validate emerging MRI techniques, determine the clinical implications of findings and explore the underlying mechanisms and biology of BMs from BC. MRI is a valuable tool for diagnosis, targeted therapy and studying BC metastasis.

## Graphical abstract

Magnetic resonance imaging (MRI) characterises and monitors brain metastases in breast cancer (BC), with findings varying by MRI technique, BC subtype, lesion features and comparison to other brain tumours. MRI is valuable for diagnosis, therapy and studying the mechanisms of BC brain metastasis.

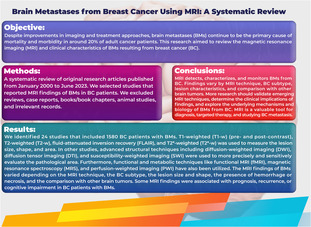

## Introduction

As a multi‐step process, metastasis involves the dissemination of cancer cells from the initial tumour to the target organ.^1^, ^2^ Brain metastases (BMs) remain the leading cause of death and morbidity in about 20% of adult cancer patients, despite advances in imaging and treatment modalities.^3^ Having BMs is a disastrous consequence for patients with solid malignancies such as breast cancer (BC), lung cancer and melanoma, and is often linked with a poor prognosis and decreased quality of life.^1^, ^4^

Because of its specialised cells, anatomical characteristics, metabolic restrictions and immunological surroundings, the brain microenvironment exerts a vastly different selection pressure on tumour cells than extracranial lesions do, which in turn influences the metastatic process and treatment responses.^5^

Approximately 15%–25% of patients with BC may develop central nervous system (CNS) metastases.^1^, ^6^ The ability of BC cells to metastasize to the brain is influenced by their receptor status. In fact, as compared to hormone receptor‐positive individuals, human epidermal growth factor receptor 2 (HER2)‐positive and triple‐negative BC (TNBC) subtypes had the highest association with CNS metastases, with the frequencies of BMs reaching values as high as 30–40%.^1^, ^7^, ^8^

Accurate identification of the cause of BMs is critical for developing an appropriate treatment plan to enhance the patients prognosis.^9^ Computed tomography (CT) scans and magnetic resonance imaging (MRI) scans are the most common imaging techniques used to diagnose brain malignancies.^10^, ^11^, ^12^, ^13^, ^14^, ^15^ The high signal‐to‐noise ratio (SNR), contrast‐to‐noise ratio (CNR), spatial resolution, contrast resolution and the abundance of MRI sequences available to define intracranial lesions allow MRI to give more detailed, localisation and characterisation of BMs.^16^, ^17^ To be more specific, T1‐weighted (T1‐w) and T2‐weighted (T2‐w) sequences outline the morphologic and anatomical de‐alignment of tissue brought about by the tumour.^18^

The purpose of this systematic review was to document the utility of reported MRI techniques in imaging BC, as well as the MRI and clinical features of BMs from BC to aid in the diagnosis and treatment of similar individuals in the future.

## Material and Methods

### Search strategy

This systematic review followed the PRISMA statement for reporting systematic reviews and meta‐analyses.^19^ The aim of this review was to synthesise the available evidence on BMs from BC using MRI. We searched PubMed and Scopus databases from January 2000 to June 2023 using the following combination of keywords: "MRI," "breast cancer," "brain metastases," and "brain metastasis." We restricted our search to articles published in English that reported MRI and clinical data on BMs in BC patients. The detailed search strategy for PubMed is provided in Table S1.

### Study selection

We selected the titles and abstracts of the retrieved records for eligibility and excluded reviews (including literature reviews, systematic reviews and meta‐analyses), case reports, books and book chapters, animal studies and other irrelevant records. We obtained the full texts of the potentially eligible records and assessed their inclusion based on the following criteria: (1) original research article (2); MRI findings of BMs in BC patients; and (3) related BC findings associated with MRI. We excluded articles that did not meet these criteria or did not provide sufficient MRI data. We also checked the reference lists of included articles for additional relevant studies. After reviewing the full texts and removing any records not related to the MRI / BC findings, we identified 86 records that may meet our inclusion criteria.

### Data extraction and quality assessment

**Figure 1:**
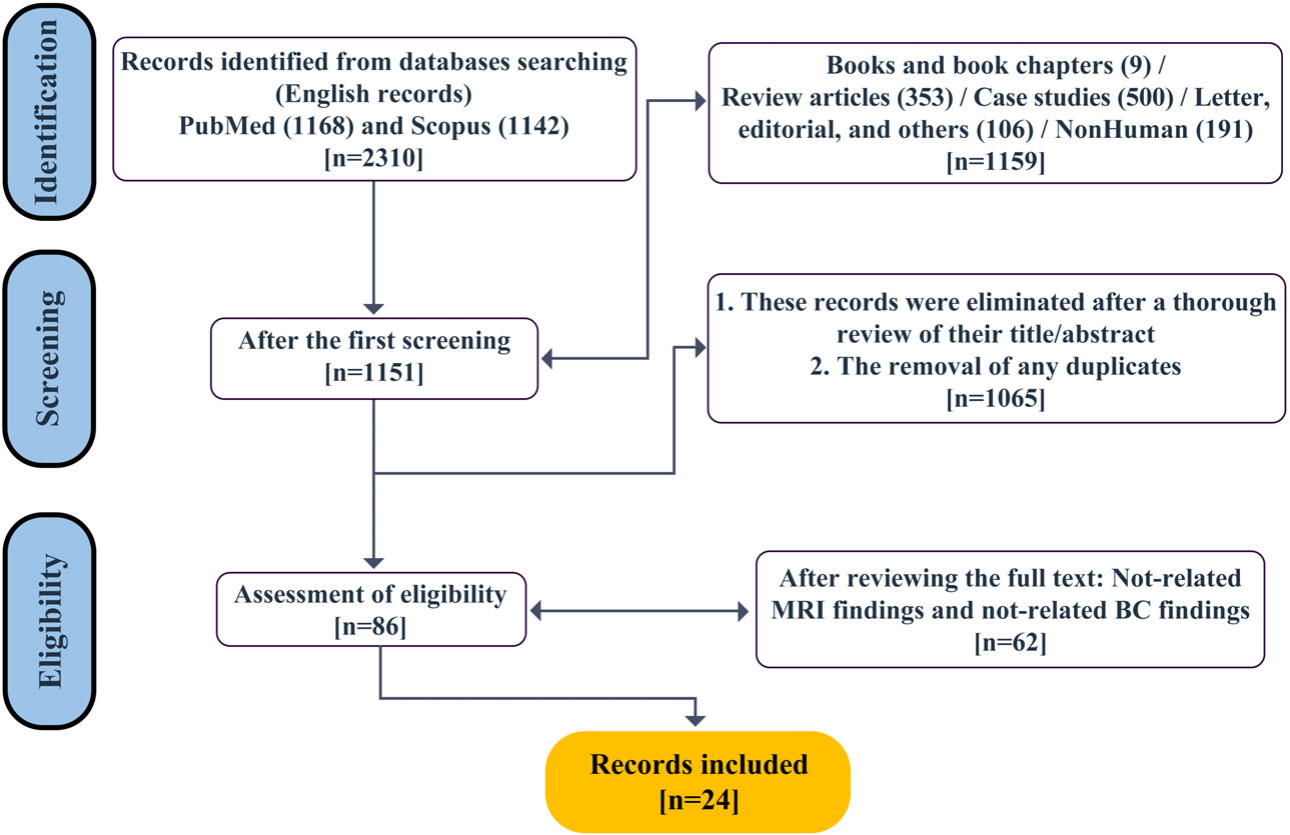
PRISMA flow diagram for systematic review.

Three independent reviewers (S.Gh., M.M. and S.M.) performed the study selection process and any disagreements were resolved by consensus or consultation. We extracted the following data from each included article: authors, year of publication, number of BC patients with BMs, MRI techniques used, anatomical locations of BMs and imaging findings. Table 1 summarises the characteristics and findings of the included studies. Other reviewers used Microsoft Excel (version 2016) to record the data. Two reviewers (S.Gh. and M.M.) independently extracted the data and cross‐checked for accuracy. Any discrepancies were resolved by discussion or consultation with a third reviewer (S.M.). The quality of the included studies was assessed using the Cochrane Risk of Bias tool. Figure 1 shows the PRISMA flow diagram of the study selection process.

**Table 1:** Brain metastases from breast cancer MRI findings.

First author (year)	Number of Patients	MRI Technique(s)	Anatomical Location(s)	MRI Findings
Xue (2023)^20^	4	T1‐w and T2‐w	Superficial parenchyma lesions and deep lesions	Following LITT, the majority of tumours exhibited an increase in volume on the MRI scan taken after 30 days. Additionally, if the EVR is greater than 40% on the 30‐day MRI, it could suggest a possibility of tumour recurrence at a later stage
Young (2023)^21^	34	CE MRI	Frontal, temporal, perietal, occipital and cerebellum	The HER2 status of BC BMs was significantly associated with lesion contour and composition on MRI
Reibelt (2022)^22^	15	3D T1‐w MPRAGE	L. cerebellum WM, R. pallidum, L. thalamus, L. choroid plexus, L. Lat. ventricle and total GM	Subcortical volume changes after radiotherapy are a sensitive indicator of neuroanatomical modifications and brain atrophy
Young (2021)^23^	38	T1‐w and T1‐w post	Frontal, temporal, perietal, occipital and cerebellum	The prospective validation of MRI enhancement for detecting HER2 overexpression in BC BMs is warranted
Santos (2020)^24^	147	T1‐w, T1‐w post, T2‐w, DWI and T2*‐w	Hippocampal and hydrocephalus	The prognosis of the disease and certain imaging features of the BM can be predicted based on the BC subtype, but not their distribution
Zhang (2019)^25^	3	T1‐w, T1‐w post, T2‐w, DWI, ADC and FLAIR	NA	ADC‐based texture analysis can be used to distinguish between solitary BM and GBM, and it is recommended that the ROI be placed on the solid portion when calculating the ADC‐based texture metrics
Mayinger (2019)^26^	851	3D T1‐w	Bilateral and central/median subcortical structures	The size of the brain's substructure varied significantly between the patients The volumetric size of the fourth ventricle could be a useful diagnostic marker in the future
Kniep (2019)^27^	37	T1‐w, T1‐w post and FLAIR	NA	Using a machine‐learning classifier trained on quantitative features of routine MR images of the brain, we found high discriminatory accuracy in predicting the tumour type of BMs
Ortiz‐Ramón (2018)^28^	17	3D T1‐w	NA	After image quantisation with the appropriate number of grey levels, the texture features of volumetric MRI can be used to distinguish between BMs and different primary cancers
Skogen (2018)^29^	5	DTI	Intracranial lesions without visible haemorrhage, multiple lesions and infratentorial lesions	Radiologists can differentiate between GBM and single BM by evaluating the heterogeneity around the tumour By having a better idea of the tumour's severity, doctors can better prepare for surgical removal and care for the patient afterwards
Muto (2018)^30^	13	DSC	Temporal lobe	DSC is a clinically useful method for distinguishing between tumour recurrence, tumour necrosis and pseudoprogression in patients with cerebral metastases A cut‐off value of 2.1 for rCBV was found to be the most accurate and consistent measurement
Kyeong (2017)^31^	100	3D T1‐w	HER2‐positive type: occipital, temporal lobes and cerebellum / Luminal type: frontal, occipital lobes and cerebellum	Hot spots of triple‐negative metastases are evenly distributed in the brain, whereas BMs of HER2 positive and luminal type occur predominantly in the occipital lobe and cerebellum When BMs from the TNBC were compared with other types, they were found more frequently in the frontal lobe, limbic region and parietal lobe Different types of BC often show a variety of BM distribution patterns
Kesler (2017)^32^	74	rsfMRI, T2*‐w GRE, HR3D IR, FSPGR GRE T1‐w and DTI	R. inferior parietal lobe, R. middle inferior orbital frontal gyrus, R. medial superior frontal gyrus, R. inferior and middle frontal gyri, bilateral postcentral gyri, R. precuneus, L. inferior temporal gyrus, L. middle occipital gyrus, R. parietal lobule, R. cuneus, R. superior temporal gyrus and R. inferior temporal gyrus.	For the BC group, structural and functional clustering was found to be significantly inversely related, while functional clustering was found to be significantly positively related to the Hurst exponent^a^ Greater cognitive impairment was associated with greater overlap between structural and functional connectome clustering The significance of structural and functional connectome properties is a potential biomarker of general neurologic deficit
Bette (2017)^33^	13	T2‐FLAIR	NA	With previously resected BMs, the FLAIR signal intensity of the fluid inside the resection cavity increases
Fan (2017)^34^	13	T1‐w FLAIR and DWI (before enhancement) and T1‐w FLAIR	R. occipital lobe	Gadobutrol (0.1 mmol/kg body weight) enhanced MR using a 3 T T1‐w FLAIR sequence, leading to more central BMs and more metastases overall. Postcontrast MRI in patients with BMs is recommended to be delayed for 7 min in clinical practice
Franceschi (2016)^35^	38	T1‐w post and SWI	NA	The occurrence of bleeding was shown to be rare in micro‐metastases using SWI and contrast‐enhanced high‐resolution T1‐w imaging, but prevalent in larger metastases independent of the initial cause, melanoma versus BC Haemorrhage was more prevalent in melanoma than in original BC, especially in the larger metastases that developed from both of these cancers
Kesler (2015)^36^	36	DTI and T1‐w	L. corpus callosum, bilateral inferior longitudinal fasciculus, L inferior fronto‐occipital fasciculus, and bilateral temporal and frontal lobe white matter	The BC group had a significantly greater number of streamline fibres than the controls, but these streamlines were shorter and had a lower mean FA
Yeh (2015)^37^	62	T1‐w, T1‐w post, T2‐w fast spin echo (FSE), FLAIR and DWI	Parietal lobe, R. frontal lobe and L. cerebellum	TNBC patients were more likely to have cystic necrotic BMs visible on MR images There are unique MRI characteristics of patients with TNBC BMs that aid in the evaluation of newly developed BMs
Quattrocchi (2014)^38^	42	T1w, T1‐w post and T2‐w FLAIR	Parieto‐occipital lobes and cerebellum	The large proportion of patients with BC and the high number of cerebellar metastases influenced the results in the non‐lung cancer group Patients showed significant clusters in the cerebellum when WMHs was absent. In contrast, no distribution shift was observed in the presence of WMH, suggesting that WMH does not have any effect on the brain distribution of BM in these patients
Huang (2010)^39^	17	Multivoxel 2D‐CSI MRS and DCE (n = 21 BC)	NA	HIF‐1 can be responsible for the association between choline metabolism and tumour perfusion in BMs
Hakyemez (2010)^40^	5	T2‐w and PWI for rCBV	NA	PWI is used as a supportive method in cases where conventional images fail to differentiate metastatic lesions from gliomas Intra‐tumoural rCBV measurements do not contribute significantly to this differentiation. However, as peritumoural oedema displays low rCBV ratios in metastatic lesions and higher rCBV ratios in high‐grade gliomas, it can be used to differentiate these two lesions Mass and rCBV ratios of the oedema surrounding the tumour prior to operation in solitary masses proved to be useful for differentiating metastases from high‐grade gliomas
Takeda (2008)^41^	13	2D T1‐w SE, 3D MPRAGE T1‐w post, 2D T1‐w SE post and 2D T2‐w SE	Posterior fossa, middle fossa and supratentorial	BM detection using 3D MPRAGE was more effective than using 2D SE
Kremer (2003)^42^	2	T1‐w, T2‐w, T2*‐w, R2* and DCE	NA	Large and solitary necrotic metastases can be indistinguishable from high‐grade astrocytomas A high rCBV level can indicate a hypervascular lesion
Geijer (2002)^43^	1	T1‐w, T2‐w, DWI and ADC	Lateral wall of the posterior horn of the L. lateral ventricle and falx cerebri	In BC metastases, the lesions were surrounded by oedema Standard DWI protocols show significant overlap between the characteristics of common BMs and those of subacute and acute ischemic lesions

2D T1‐w SE, two‐dimensional T1‐weighted spin echo; 2D T2‐w SE, two‐dimensional T2‐weighted spin echo; 3D T1‐w MPRAGE, 3D T1‐weighted magnetisation prepared rapid acquisition gradient echo; ADC, apparent diffusion coefficient; BC, breast cancer; BMs, brain metastases; CBV, cerebral blood volume; CE MRI, contrast‐enhanced MRI; CE, contrast‐enhanced; DCE, dynamic contrast‐enhanced; DSC, dynamic susceptibility contrast; DTI, diffusion tensor imaging; DWI, diffusion‐weighted imaging; EVR, enhanced volume ratio; FLAIR, fluid‐attenuated inversion recovery; FSPGR, fast spoiled gradient recalled; GBM, glioblastoma multiforme; HER2, human epidermal growth factor receptor 2; HIF‐1, hypoxia‐inducible factor‐1; HR3D IR high‐resolution 3D inversion recovery; L., Left; LAVA, liquid attenuation inversion recovery; LITT, laser interstitial thermal therapy; MPRAGE, magnetisation prepared rapid acquisition gradient echo; MRI, magnetic resonance imaging; MRS, magnetic resonance spectroscopy; Multivoxel 2D‐CSI MRS, multivoxel two‐dimensional chemical shift Imaging magnetic resonance spectroscopy; PWI, perfusion‐weighted imaging; R., right; R2*, transverse relaxation rate; rCBV, relative cerebral blood volume; rsfMRI, resting‐state functional MRI; SWI, susceptibility‐weighted imaging; T1‐w post, T1‐weighted postcontrast; T1‐w, T1‐weighted; T2*‐w, T2‐star weighted; T2‐w, T2‐weighted; TNBC, triple‐negative breast cancer; WMH, white matter hyperintensities.

^a^ Hurst exponent measures a time series' memory, with values >0.5 showing trends and <0.5 indicating mean reversion.

## Results

Twenty‐four studies that investigated BMs from BC were included in the analysis (Table 1). The studies included a total of 1580 patients with BMs from BC and used various MRI techniques such as T1‐w, T2‐w, contrast‐enhanced (CE), diffusion‐weighted imaging (DWI), fluid‐attenuated inversion recovery (FLAIR), dynamic susceptibility contrast (DSC), resting‐state functional MRI (rsfMRI), susceptibility‐weighted imaging (SWI), magnetic resonance spectroscopy (MRS) and perfusion‐weighted imaging (PWI). The anatomical locations of BMs varied across studies, but some common regions were the frontal, temporal, parietal and occipital lobes, the cerebellum and the ventricles. The MRI findings of BMs also varied depending on the MRI technique, the BC subtype, the lesion size and shape, the presence of haemorrhage or necrosis and the comparison with other brain tumours such as glioblastoma multiforme (GBM). Some MRI findings were associated with prognosis, recurrence or cognitive impairment in BC patients with BMs. The table provides a comprehensive overview of the current state of knowledge on BMs from BC using MRI.

The MRI findings for BMs from BC based on the studies in Table 1 indicate that the most common location for metastases is in the cerebral hemispheres. The majority of metastases are solid‐enhancing masses with irregular or poorly defined margins. The number of patients in each study ranged from 1 to 851. The enhancement pattern of BMs was reported to be variable, with some studies reporting homogeneous enhancement, while others reported heterogeneous or rim enhancement.

## Discussion

The aim of this systematic review was to summarise the MRI techniques, anatomical locations and MRI findings of BMs from BC using different MRI modalities. The MRI findings from these studies can be broadly categorised into lesion identification, characterisation and prediction of prognosis or response to treatment.

MRI is a useful and versatile tool for detecting, characterising and monitoring BMs from BC, as it can provide information on the lesion size, shape, location, composition, perfusion, metabolism and connectivity.^26^, ^32^, ^41^ MRI techniques can vary in their sensitivity and specificity for BMs from BC, depending on the contrast agent, the sequence parameters, the image processing and the interpretation criteria.^21^, ^34^, ^35^ Some MRI techniques such as CE MRI, DWI, ADC, DSC and SWI can help to differentiate BMs from BC from other brain tumours such as GBM or ischemic lesions.^29^, ^30^, ^34^, ^35^, ^40^, ^42^ Other MRI techniques such as postcontrast T1‐w and FLAIR can help to assess the response to treatment or the recurrence of BMs from BC.^33^, ^34^, ^35^ Some MRI techniques such as DTI and rsfMRI can help to evaluate the cognitive impairment or the functional connectivity of BMs from BC.^29^, ^32^, ^36^

MRI findings of BMs from BC can vary depending on the BC subtype, such as HER2‐positive, luminal or triple‐negative. Different BC subtypes can have different patterns of BM distribution in the brain, different lesion contours and compositions on MRI, and different associations with prognosis or imaging features. For example, HER2‐positive BMs tend to occur in the occipital lobe and cerebellum and have a smooth contour and a solid composition on MRI. Triple‐negative BMs tend to be evenly distributed in the brain and have a lobulated contour and a cystic necrotic composition on MRI.^21^, ^23^, ^31^, ^37^

MRI findings of BMs from BC can also vary depending on the lesion size and shape, the presence of haemorrhage or necrosis and the comparison with other brain tumours such as GBM or ischemic lesions. Larger BMs tend to have more haemorrhage and necrosis than smaller BMs. Haemorrhage is more prevalent in melanoma than in BC BMs. Necrosis is more common in GBM than in BMs.^30^, ^35^ ADC‐based texture analysis can help to distinguish between solitary BM and GBM by measuring the heterogeneity of the lesion.^25^ rCBV can help to distinguish between metastatic lesions and gliomas by measuring the perfusion of the lesion and the surrounding oedema.^40^, ^42^

MRI techniques have also been used to predict prognosis and treatment response in BC BMs.^24^ Xue et al. observed an increase in tumour volume on MRI scans following laser interstitial thermal therapy (LITT) and suggested that an enhanced volume ratio (EVR) greater than 40% on the 30‐day MRI could indicate a potential for tumour recurrence.^20^ Reibelt et al. found that subcortical volume changes after radiotherapy were sensitive indicators of neuroanatomical modifications and brain atrophy,^22^ while Muto et al. demonstrated the clinical utility of DSC in distinguishing between tumour recurrence, tumour necrosis and pseudoprogression in cerebral metastases.^30^ These findings underscore the potential of MRI in monitoring treatment response and predicting outcomes in BC BM patients.

The strengths of this review are that it provides a comprehensive overview of the current state of knowledge on BMs from BC using MRI. It covers a wide range of MRI techniques, anatomical locations and MRI findings that are relevant for clinical practice and research. It also identifies some knowledge gaps and limitations that need further investigation.

This review has some limitations that should be acknowledged and addressed in future research. The studies included in the analysis may have been subject to selection bias, as they were not randomly selected and may not represent the broader population of patients with BMs. The patients included in the studies varied in terms of tumour type, size and location, as well as demographic and clinical characteristics. This heterogeneity could introduce variability into the findings and limit the generalisability of the results. The studies used different MRI techniques and protocols, which could affect the accuracy and reliability of the findings. The studies used different criteria for defining and classifying MRI findings, which could lead to inconsistencies and difficulties in comparing results across studies. Some studies had limited follow‐up periods, which could limit the ability to assess longer‐term outcomes and the recurrence of BMs.

Based on the findings and limitations of this review, some recommendations for future research can be made. First, more studies are needed to validate some MRI techniques for BMs from BC, such as ADC‐based texture analysis, rCBV measurement, machine‐learning classifier, and structural and functional connectome analysis. These techniques have shown promising results in distinguishing BMs from BC from other brain tumours or predicting their prognosis or cognitive impairment, but they need further confirmation and refinement in larger and more diverse samples. Second, more studies are needed to explore the clinical implications of MRI findings for BMs from BC, such as their impact on treatment decision‐making, survival outcomes, quality of life or functional recovery. These studies can help to translate the MRI findings into meaningful and actionable information for patients and clinicians. Third, more studies are needed to investigate the underlying mechanisms and pathways of BMs from BC using MRI, such as their molecular and genetic characteristics, their interaction with the brain microenvironment, or their response to therapy. These studies can help to elucidate the pathophysiology and biology of BMs from BC and identify potential targets or biomarkers for diagnosis or treatment.

## Conclusion

Diseases may be diagnosed earlier and more precisely with the use of medical imaging, especially MRI. This review shows that MRI is a valuable tool for detecting, characterising and monitoring BMs from BC using different MRI techniques. MRI can be used to examine the structure, function, metabolism and important MRI biomarkers. MRI can provide information on various aspects of BMs from BC such as their size, shape, location, composition, perfusion, metabolism and connectivity. MRI findings of BMs from BC can vary depending on the MRI technique, the BC subtype, the lesion size and shape, the presence of haemorrhage or necrosis, and the comparison with other brain tumours.

MRI can aid in the early diagnosis of tumours and their spread in the initial phases of their growth and metastasis. In addition to its better sensitivity, MR imaging's unique characteristics (such as its high SNR, CNR, spatial resolution and contrast resolution) make it a valuable tool for prognosis and early identification of disease, all of which are critical for implementing targeted therapy. Further studies are needed to validate some MRI techniques for BMs from BC and to explore their clinical implications.

## Conflict of Interest

The authors declare no financial or other conflicts of interest.

## Supporting information


**Table S1.** Search strategy and MeSH Terms on PubMed databases.

## Data Availability

The data that support the findings of this study are available on request from the corresponding author.
