# Supplementary material for: Brain metastases from breast cancer using magnetic resonance imaging: A systematic review
Source: J Med Radiat Sci. 2023 Aug 10;71(1):133–41. doi: 10.1002/jmrs.715 (PMC10920938; doi:10.1002/jmrs.715)
Supplement: Supplementary file 1 — Table S1. Search strategy and MeSH Terms on PubMed databases. [file JMRS-71-133-s001.docx]

| **Supplementary Table 1.** Search strategy and MeSH Terms on PubMed databases | |
| --- | --- |
| Keywords | Search Strategy |
| Search: (Magnetic resonance imaging OR MRI) AND (breast cancer) AND (brain metastases OR brain metastasis) AND (2000:2023[pdat])  Filters: English Sort by: Publication Date | - MRI: "magnetic resonance imaging"[MeSH Terms] OR ("magnetic"[All Fields] AND "resonance"[All Fields] AND "imaging"[All Fields]) OR "magnetic resonance imaging"[All Fields] OR "mri"[All Fields] - breast cancer: "breast neoplasms"[MeSH Terms] OR ("breast"[All Fields] AND "neoplasms"[All Fields]) OR "breast neoplasms"[All Fields] OR ("breast"[All Fields] AND "cancer"[All Fields]) OR "breast cancer"[All Fields] - brain metastases: "brain neoplasms"[MeSH Terms] OR ("brain"[All Fields] AND "neoplasms"[All Fields]) OR "brain neoplasms"[All Fields] OR ("brain"[All Fields] AND "metastases"[All Fields]) OR "brain metastases"[All Fields] - brain metastasis: "brain neoplasms"[MeSH Terms] OR ("brain"[All Fields] AND "neoplasms"[All Fields]) OR "brain neoplasms"[All Fields] OR ("brain"[All Fields] AND "metastasis"[All Fields]) OR "brain metastasis"[All Fields] |
